# Enhancing fertility of the marine copepod *Bestiolina amoyensis* via multi-generational selective breeding

**DOI:** 10.1038/s41598-025-33672-6

**Published:** 2026-01-05

**Authors:** Zhouyang Ma, Run Hu, Shuhong Wang, Nan Chen

**Affiliations:** 1 State Key Laboratory of Mariculture Breeding, Fisheries College of Jimei University, Yindou Road 43, Xiamen, 361021 Fujian China; 2 Ornamental Aquarium Engineering Research Center in University of Fujian Province, Yindou Road 43, Xiamen, 361021 Fujian China; 3 Key Laboratory of Healthy Mariculture for the East China Sea, Ministry of Agriculture and Rural Affairs, Xiamen, 361021 China

**Keywords:** Genetic breeding, Live feed, Calanoid copepods, Total egg production, Agricultural genetics, Animal breeding, Marine biology

## Abstract

Copepods are a group of marine plankton that play an essential role in the energy transfer within marine ecosystems. They serve as vital prey for the larvae of numerous economically important fish, shrimp, and crabs species. *Bestiolina amoyensis*, a copepod species inhabiting nearshore warm waters, possesses numerous commercial advantages for aquaculture due to its small size, rapid reproduction, and strong adaptability, that make it well-suited for use as live prey in aquaculture systems. In this study, a breeding program targeting high fertility was designed and implemented*.* Fifty pairs of adult *B. amoyensis* were randomly selected to establish the base population (G0), with the total egg production used as the breeding index. In the selected group, offspring from maternal lines that ranked in the top 30% for total egg production were selected and eggs laid on the 4^th^ day by these selected pairs were used to establish breeding pairs for subsequent generations. In the control group, breeding pairs were established by randomly mating offspring of the G0 population. The selection procedure was repeated for five consecutive generations, with consistent selection criteria maintained only for the selected group. Additionally, the stability of selection effect was investigated by tracking both groups for an another 5, 10, 15 generations after the termination of artificial selection. After five generations of selection, the selected group showed a significant increase in total egg production, whereas no significant change was observed in the control group. By the fifth generation (G5), the selected group exhibited a 23.99% increase in total egg production compared with the control group, with a heritability (h^2^) of 0.46 ± 0.11. At the G5 generation, the population densities of the selected and control groups were 9075.00 ± 193.18 ind/L and 7200.33 ± 249.18 ind/L, respectively. After rearing for another 5, 10, and 15 generations without selection, the total egg production of the selected groups was 167.50 ± 2.47 eggs/female, 164.23 ± 2.46 eggs/female and 158.23 ± 3.14 eggs/female, respectively, indicating a gradual decrease. However, the selected group maintained significantly higher egg production than the control group (*p* < 0.05). The results demonstrate that the reproductive capacity of *B. amoyensis* can be enhanced through continuous selection, providing technical support for the large-scale and high-density cultivation of this copepod species.

## Introduction

Copepods, a group of oceanic zooplankton, form a key link between primary producers and secondary consumers in marine ecosystems^1^. Copepods constitute the primary diet item for many planktivorous organisms at higher trophic levels and are the preferred prey of various developmental stages of economically important species of fish and shrimp^2^. The copepods utilized as live feed in aquaculture include Calanoida, Cyclopoida, and Harpacticoida. Calanoid copepods, abundant in the upper and middle layers of nearshore waters, produce relatively small nauplii with a complete planktonic life cycle, making them the most suitable initial feed for fish larvae in the planktonic stage^1^.

Marine copepods are rich in highly unsaturated fatty acids (HUFA)^3,4^, including DHA and EPA, and their fatty acid content is significantly higher than that of nutritionally fortified rotifers and artemia^5^. Notably, the DHA/EPA ratio in copepods exceeds 1, offering greater benefits for the growth and development of predators. Moreover, many newly hatched fish, due to their small mouth sizes, struggle to consume larger foods such as rotifers and artemia^6^. The early nauplii of small copepods, being less than 80 µm in size, are ideally suited for fish with small mouths^7^. Additionally, copepods exhibit a unique swim-and-sink motion, with nauplii primarily moving by jumping and copepodites and adults through intermittent jumping, triggering strong feeding responses in fish larvae^8^. Research has demonstrated the success of using copepod nauplii as initial feed in cultivating marine species such as *Epinephelus coioides*^9,10^, *Centropyge loriculus*^11^, *Trachinotus carolinus*^12^, *Rivulus marmoratus*^13^, *Hippocampus subelongatus*^14^, *Glaucosoma hebraicum*^15^ and *Zebrasoma flavescens*^16^*.* Thus, copepods represent a promising live feed option for aquaculture.

Previous research on the cultivation of copepods has focused on external culture conditions, such as temperature^17,18^, salinity^19,20^, light^21–23^, and food^24–26^. Appropriate conditions enable to achieve the maximum productivity of copepods. By contrast, relatively few studies have been conducted on the improvement of copepods’ production traits.

Notably, copepods have a shorter generation cycle than large aquatic animals such as fish and shrimp, which makes it an ideal organism for the study of selective breeding^27–29^ and multigenerational acclimatisation^30^. The potential of selection to improve copepods’ production-related traits has been demonstrated in several studies. For example, Alajmi et al.^31^ focused on egg production of *Parvocalanus crassirostris* on the third and fourth days, and egg production on those days increased by 8% (G1) to 20% (G5) after five generations of selection. Souissi et al.^32^ conducted temperature selection on *Eurytemora affinis* for several generations at 7 °C and 20 °C. The results showed that the body length and fatty acids contents of copepods reared at 7 °C were significantly higher than those of copepods cultured at 20 °C. After several generations of cultivation at 24 °C, these advantages in body length and fatty acid contents persisted under low temperatures^32^. Santhanam et al.^33^ conducted a five-generation selection experiment on the egg production of *Pseudodaptomus annandalei* at 18 °C and 26 °C. By the fifth generation, the gain in egg production increased by 29.60% at 26 °C, and 31.90% at 18 °C. At 26 °C, the population size of the selected group increased by 42.81% compared to that of the control group^33^. Additionally, a high-astaxanthin dark-body strain (DBS) strain of *Pseudodiaptomus annandalei* strain was established through selective breeding. This selected strain has a significantly higher astaxanthin content than that reported in other copepod species^34^. However, whether the genetic gains observed in these studies would be lost during subsequent rearing has not yet been reported.

*Bestiolina amoyensis*, a copepod species inhabiting nearshore warm waters, possesses numerous economic advantages, including small size, rapid reproduction, and strong adaptability. These traits make it a potential a promising live feed species^35,36^.

The confirmation of target traits is crucial for research in selective breeding, as it directly determines the direction of population selection. Compared to daily egg production, total egg production can comprehensively reflect the reproductive capacity of females, while daily egg production only represents the number of eggs produced on a single day. Research has shown that daily egg production varies significantly during the entire reproductive cycle^37^. The reproductive lifespan and hatching rate of females vary relatively little among individuals under suitable culture conditions.

Therefore, this study aims to improve the total egg production of *B. amoyensis* through artificial selection. First, a comprehensive study focusing on the total egg production was conducted. Then, fifty pairs of adult *B. amoyensis* were randomly selected to establish the base population (G0). Subsequently, distinct breeding protocols were implemented for the selected and the control group, respectively. For the selected group, offspring representing the top 30% in egg production of G0 were chosen, and the eggs laid on the 4^th^ day were used to establish G1. This selection intensity (targeting the top 30% in egg production) was consistently maintained and applied to the breeding of G2 to G5, all of which constituted the selected group. To thoroughly assess the impact of artificial selection, comparisons between selected and control groups were continued for the 5th, 10th, and 15th generations after the cessation of selection pressure in the selected group. If the results demonstrate that artificial selection can achieve a stable improvement in total egg production, this would provide the possibility of increasing the culture density of *B. amoyensis* or sustainably and efficiently obtaining live feed, thereby indicating its great potential for large-scale cultivation.

## Materials and methods

### Microalgal culture

The microalga *Isochrysis galbana* used in this study was obtained from the Collaborative Innovation Centre of Ornamental Marine Species and Public Service Platform of Industrialization (Xiamen, China). The algae were grown in 70 L cylindrical acrylic tanks using seawater that was filtered to 0.01 µm, UV-irradiated, and chlorinated (salinity of 28 psu). The microalgae were kept at a constant 25 ± 1 °C under gentle aeration. The cultures were grown on f/2 medium following Ryther and Guillard^38^ under a continuous 24 h light cycle with an intensity of approximately 4000–5000 lx. The feed concentration was standardized. Microalgae in exponential growth phase were used in all experiment and five samples were measured daily using an automated cell counter to maintain the designated concentration (about 1 × 10^5^ cells mL^−1^).

### *Bestiolina amoyensis* stock culture

Copepods were harvested in July 2022 using a plankton net from Wuyuan Bay, Huli District, Xiamen Province, China, and subsequently transported to the lab. Following separation^35^, the cultivation of *B. amoyensis* was carried out in a 300 L tank filled with seawater filtered to 0.01 μm and supplied with gentle aeration. The culture conditions were maintained at a salinity of 28 ± 1 psu, a temperature of 26 ± 1 °C, and a photoperiod of 12 h of light followed by 12 h of darkness, with a light intensity of 500 lx. All the experiments in this article were conducted under the above-mentioned culture conditions. Approximately 30% of the tank water was replaced daily using a 5 cm diameter cylindrical isolation device with a 48 µm mesh bottom. Before starting the experiments, *B. amoyensis* were fed daily with *I. galbana* at a concentration of 3 × 10^5^ cells/ml. To minimize population stress, the copepods were transferred to a new tank every 15–20 days.

### Experimental design and setup

#### Spawning characteristics of *Bestiolina amoyensis*

In all experiments, mature *B. amoyensis* were collected using a specialized 500 μm sieve from a stock culture and immediately placed into a 2.5 L container with the bottom covered by a 75 μm mesh. This container was then positioned inside a larger, 5 L transparent vessel filled with 4 L of 0.01 μm filtered seawater. This setup was designed to easily separate adult copepods from their eggs. The eggs spawned by the females were incubated in the same 5 L container until they reached the copepodite V stage (CV). The method for obtaining the same batch of adults refers to the research of Wang et al. (2021)^35^. These late-stage copepodites were transferred to 30 mL Petri dishes for daily monitoring under a microscope to follow their molting progression until they matured into adults, at which time they were prepared for subsequent experiments. The conditions for these experiments followed established culture conditions (Sect. "*Bestiolina amoyensis* stock culture"). Thirty treatment groups were established to study egg production and the reproductive lifespan of females. Active and intact adult pairs of *B. amoyensis* were selected from a 5 L beaker and carefully moved to individual 30 mL Petri dishes filled with 20 mL of seawater for observation of daily egg production. The reproductive lifespan of females was determined after all copepod groups had ceased spawning. During the study, each pair of copepods was transferred daily to a new Petri dish with fresh seawater, and microalgae were provided at a specific concentration every 24 h.

Thirty treatment groups were similarly established for the examination of egg hatching rates. Adult copepods were obtained as described in Sect. "*Bestiolina amoyensis* stock culture". Eggs were collected every three hours and transferred to new Petri dishes. Incubation conditions were set at 26 °C with a light intensity of 0 lx for six hours, following the methodology described by Wang et al.^38^. The daily egg hatching rate was recorded until spawning ceased across all copepod groups. The hatching rate was calculated as follows:

$${\text{The daily egg hatching rate}} = \frac{h}{e} \times 100,$$

where e represents the daily egg production of each pair; h represents the number of hatched nauplii.

#### Breeding selection

The selection approach of Alajmi et al.^31^ was adapted with minor modifications, specifically that only the offspring produced on the 4^th^ day was selected as the female parents for breeding the next generation, and the total egg production of *B. amoyensis* was used as the selection index.

**Fig. 1:**
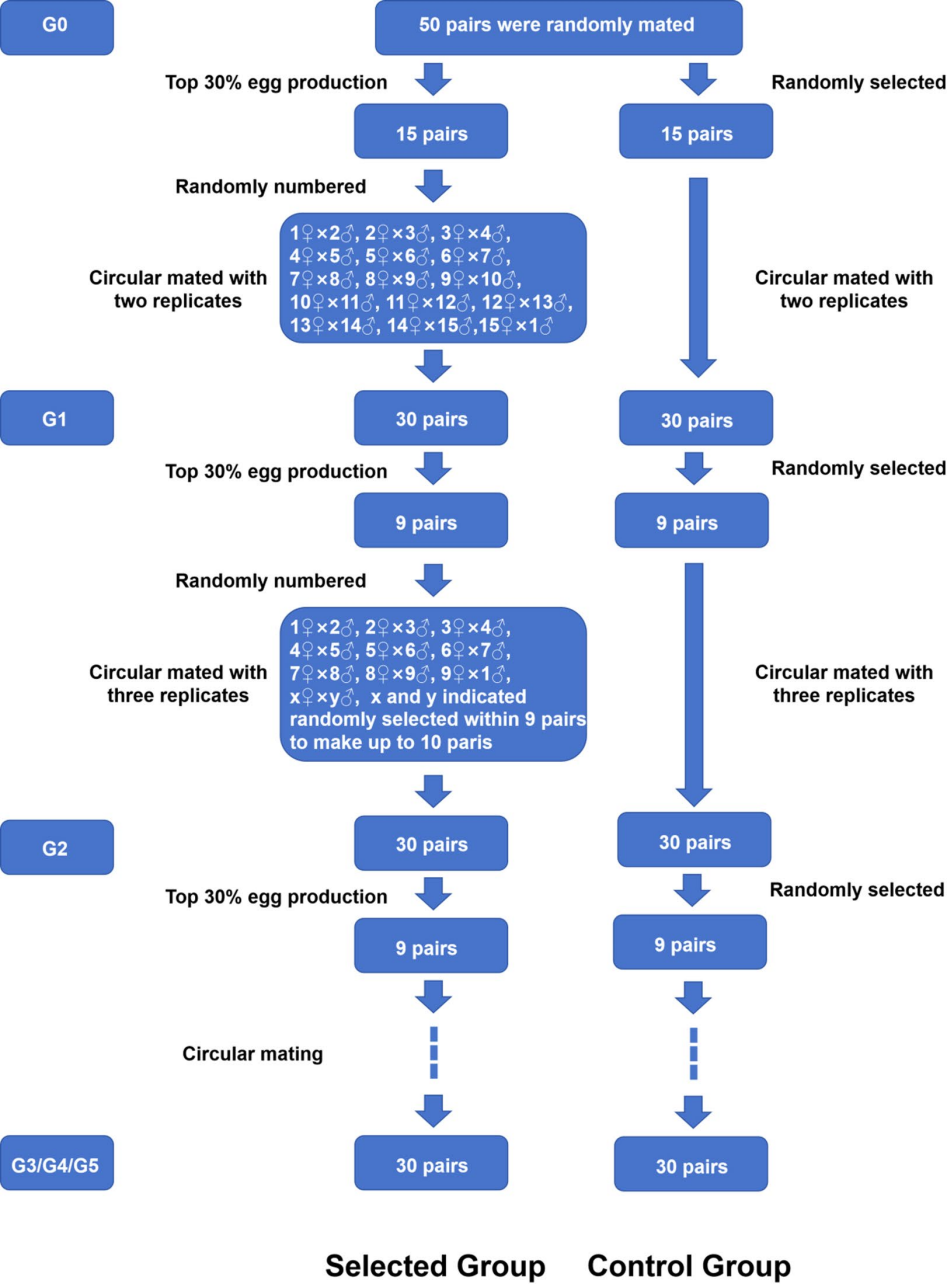
Protocol of breeding strategy for selected group and control group based on the total egg production.

Fifty pairs of adult *B. amoyensis* (sourced from the population described in Sect. "Spawning characteristics of* Bestiolina amoyensis*") exhibiting active swimming and intact appendages were randomly chosen to establish the base population (G0). The daily egg production of each G0 female was monitored, and total egg production until cessation of spawning was recorded. The offspring representing the top 30% in egg production were used to form 30 G1 pairs through circular mating involving different parents for the selected group, and 30 G1 pairs formed by random mating comprised the control group (Fig. 1). This selection process was repeated for five generations, maintaining the same criteria for the selected lines. For each new generation, a control line was also maintained, following the procedure outlined for G1.

For each pair, the male and female were transferred to the 30 mL Petri dish filled with 20 mL seawater. After the female individual began spawning, the parents individuals were transferred to a new Petri dish daily, while the laid eggs were retained in the original dish. This serial relocation protocol would be sustained until reproductive cessation, thereby facilitating the precise documentation of daily fecundity metrics for each breeding pair and collection of eggs produced on fourth day. To mitigate environmental confounding variables, environmental conditions, as delineated in Sect. "*Bestiolina amoyensis* stock culture" (salinity: 28 ± 1 psu, temperature: 26 ± 1 °C, photoperiod: 12:12 light and dark), were rigorously standardized and consistent across all breeding pairs and temporal phases of the investigation.

The parallel experiments were conducted to study copepod population dynamics. Each group of experiments was repeated three times. Eight females and four males from the fifth-generation offspring of both the selected and control groups were randomly selected and cultured in a 1 L beaker for 12 days. Thirty percent of the culture water was changed every 24 h. After 12 days, all the cultures were fixed and the number of eggs, nauplii, copepodites, and adults were counted to assess the population density and structure. Population size refers to the number of individuals at all stages of development. The intrinsic growth rate was calculated using the following formula:

$${\mathrm{R}} = {\mathrm{ln}}\left( {{\mathrm{N}}1/{\mathrm{N}}0} \right)/{\mathrm{t}},$$

where N0 is the initial population size (in this experiment, the initial population size was 12, comprising eight females and four males); N1 is the final population size (the sample size included all life stages after 12 days was assessed via direct counting), and t is the number of days of the experiment.

Genetic gain refers to the level to which a certain trait has been improved in offspring through selection and breeding^39^. The difference in mean phenotype (total egg production) between the progeny generation and the previous generation is used to determine the response (R). The selection differential (S) is calculated based on the mean phenotypic difference between the chosen parents and the overall parent population. The realized heritability (h^2^) is estimated from the slope of the regression line of generation means plotted against the cumulative selection differential^40^. Genetic gain (G) achieved over two consecutive generations of selection was calculated using the equation:

$${\text{G }} = {\text{ }}({\mathrm{Sn}} - {\mathrm{Cn}})/{\mathrm{Cn}},$$

where Sn is the mean phenotypic value of the select group, and Cn is the mean phenotypic value of the control group; n represents the number of generations of selection.

#### Estimating the stability of the selective effect

To verify whether the enhanced egg-laying capacity would be rapidly lost upon the removal of external selective pressure, the reproductive capacity of both the selected group and the control group was tracked over 5, 10, and 15 generations without artificial interference. The offspring produced on the fourth day of G5 (all 30 pairs, nearly 1000 eggs) were collected and transferred to a new 10 L tank, where they were reared under the conditions described in Sect. "*Bestiolina amoyensis* stock culture" to establish the F0 generation. Individuals of the F0 generation were allowed to mate freely in culture tanks and maintained under careful husbandry. Once females began releasing eggs, the parental individuals were relocated to a new tank daily, while the eggs laid would remain in the original tank. 1000 eggs produced on the fourth day were randomly selected and pooled to form the F1 generation. Following this protocol, the 1000 offspring produced on the fourth day of each subsequent generation were collected for continuous cultivation until samples of the F15 generation were obtained.

The total egg production and population dynamics for the selected and control groups were measured at the F5, F10 and F15 generations. For the assessment of total egg production, 30 matured pairs were randomly selected from the F5, F10 and F15 tanks and incubated separately until spawning ceases, following the methodology described in Sect. "Spawning characteristics of *Bestiolina amoyensis*". For population dynamics, the experiment was conducted with 3 replicates, each replicate contained eight females and four males within F5, F10 and F15 samples, which were placed in 1L beakers for 12 days, in accordance with the methodology outlined in Sect. "Breeding selection". For clearer differentiation, individuals derived from parents in the control group were designated as F, whereas those originating from the selected group were designated as F’.

### Data analysis

The differences in egg production, reproductive lifespan, population density, and intrinsic growth rate between the selected group and the control group, as well as differences in egg production among generations were analyzed using one-way ANOVA, and multiple comparisons were conducted with Tukey’s HSD test. Heritability was analyzed using linear regression. All statistical analyses were conducted using IBM SPSS Statistics 26 software, and data are presented as mean ± standard error (SE). A significant difference was defined as *p* < 0.05.

## Results

### Spawning characteristics of *Bestiolina amoyensis*

**Fig. 2:**
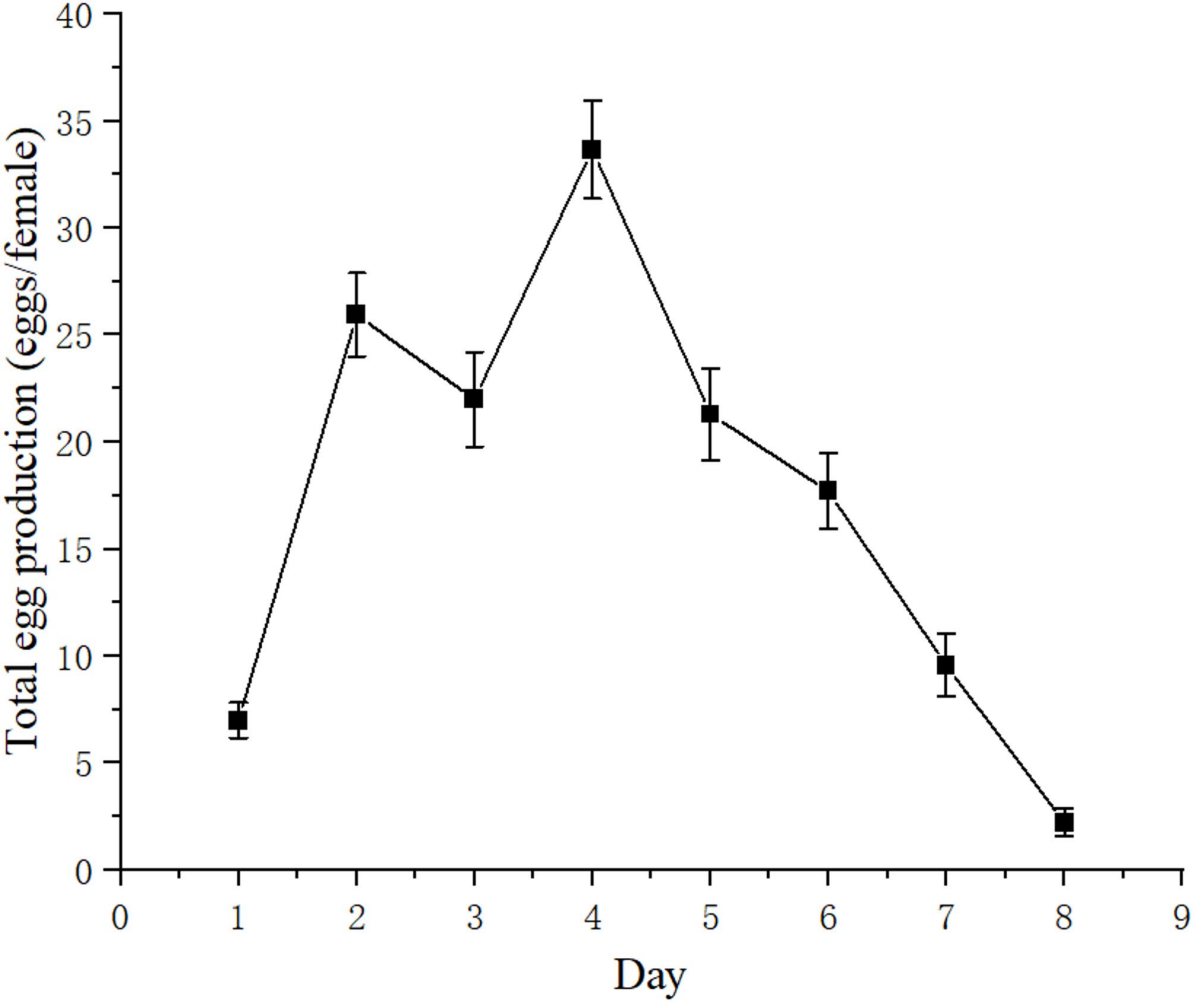
Daily egg production of *Bestiolina amoyensis* during the female reproductive period (n = 30).

**Fig. 3:**
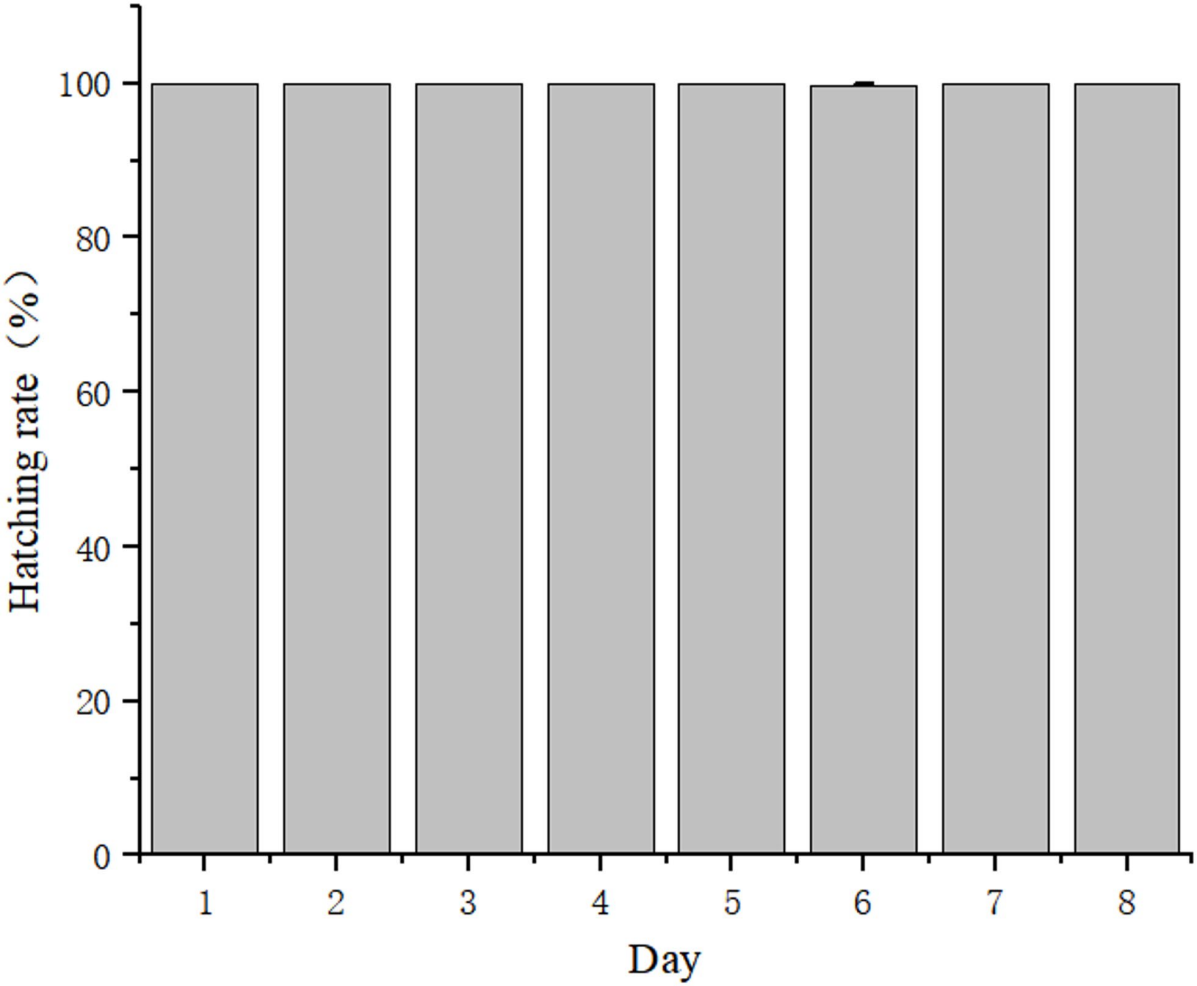
Daily egg hatching rate of *Bestiolina amoyensis* during the female reproductive period (n = 30).

As shown in Fig. 2, the daily egg production of *B. amoyensis* was recorded. Daily egg production peaked on the 4^th^ day, with an average of 33.67 ± 2.30 eggs/female. After this peak, daily egg production gradually decreased until the 8^th^ day, and all females ceased spawning by the 9^th^ day. The total egg production during the entire reproductive period was 139.70 ± 4.40 eggs/female. There was no significant difference in the egg-hatching rate of *B. amoyensis* across the female reproductive lifespan (*p* > 0.05). The hatching rate was 99.70% on the sixth day and 100% on other days (Fig. 3).

### Breeding selection

**Fig. 4:**
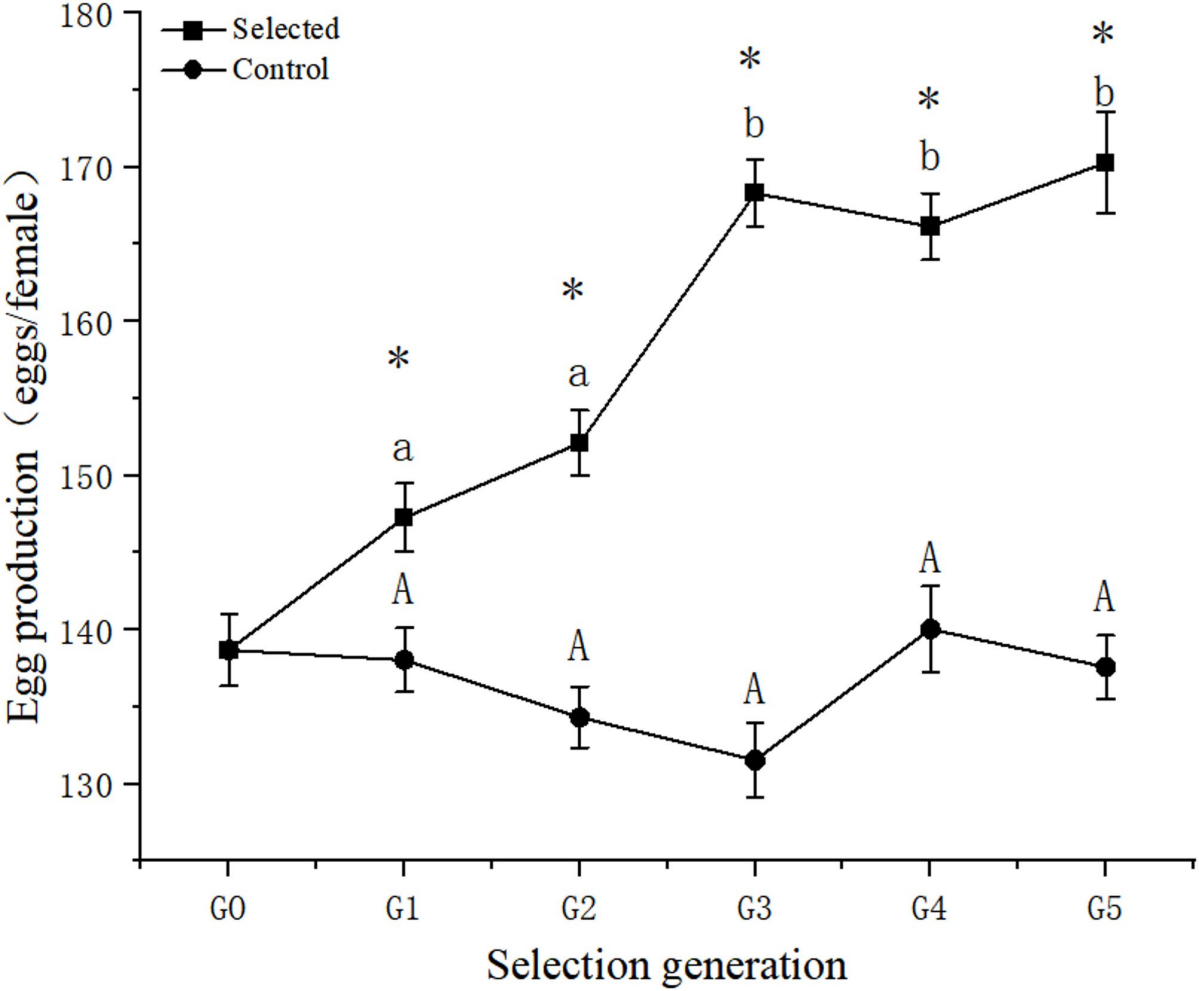
Total egg production (eggs/female) of *Bestiolina amoyensis* of the selected group and the control group across five generations of selection. The “*” indicates that a significant difference was observed between the selected and control group. Different letters denote significant differences among the generations.

**Fig. 5:**
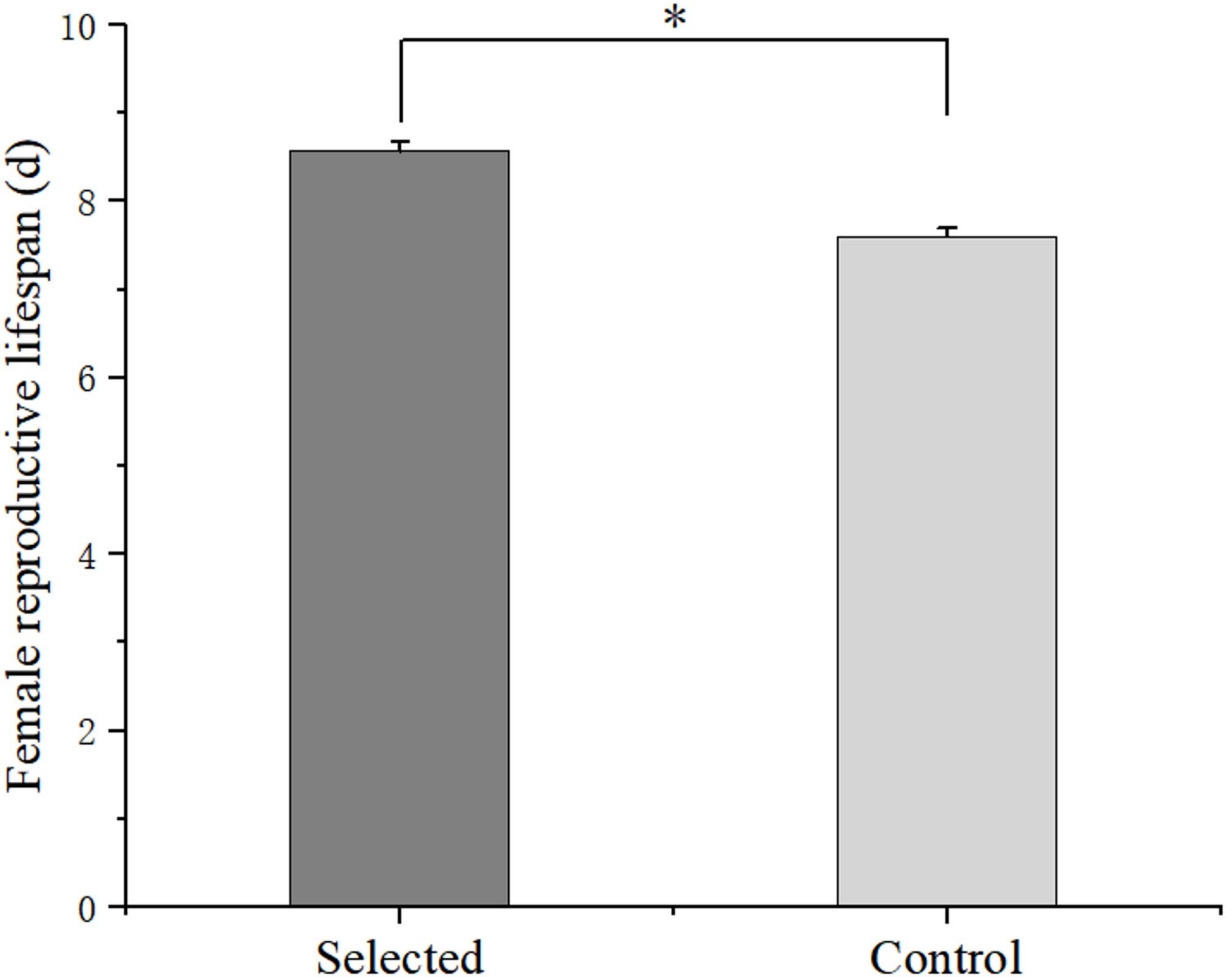
Female reproductive lifespan between the selected group and the control group across five generations of selection. Data are presented as mean ± standard error. The “*” indicates that a significant difference was observed between the selected and control groups.

After five generations of selection, there were no significant differences in total egg production of the control group (*p* > 0.05, Fig. 4). For the selected groups, total egg production of the G1 and G2 groups was significantly lower than that of the G3, G4, and G5 selected groups, and reached a peak in the G5 group (170.57 ± 3.29 eggs/female). The total egg production of the selected groups was significantly higher than that of the control group across five generations. The total egg production of the G5 selected group was significantly higher than that of the control group (137.57 ± 2.05 eggs/female). Moreover, the reproductive lifespan of females in the selected group (8.57 ± 0.11 d) was extended by 0.97 days compared to the control group (7.60 ± 0.10 d) (*p* < 0.05) (Fig. 5).

**Fig. 6:**
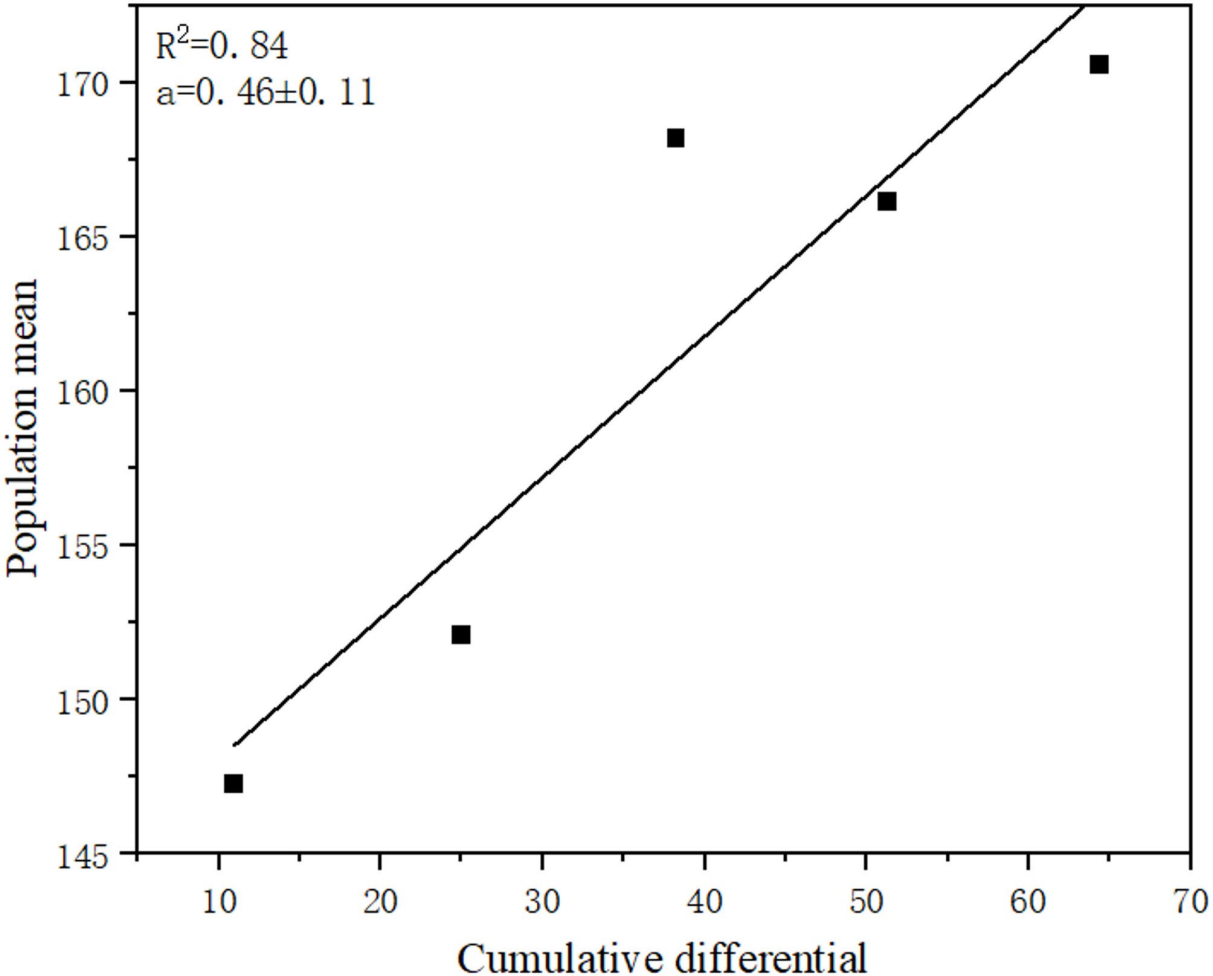
Regression between the population egg production means (eggs/female) across five generations and the cumulative selection differential.

Throughout five generations of selection, the genetic gain in total egg production increased from 6.69% in the G1 group to 23.99% in the G5 group (Table 1), while the selection differential remained constant. The heritability (h^2^) of total egg production was estimated as 0.46 ± 0.15 (Fig. 6).

**Table 1:** Genetic gain of egg production of *Bestiolina amoyensis* after five generations of selection.

Selection generations	Selected population	Selected parents	Control	R (S_n_ – C_n_)	G(%)	*Selection differential
G_0_	138.68 ± 2.35	149.63 ± 1.59	–	–	–	10.95
G_1_	147.27 ± 2.22^a^	161.33 ± 2.64	138.03 ± 2.07^b^	9.24	6.69%	14.06
G_2_	152.10 ± 2.11^a^	165.33 ± 1.82	134.43 ± 1.99^b^	17.67	13.14%	13.23
G_3_	168.20 ± 2.16^a^	181.22 ± 1.50	131.53 ± 2.41^b^	36.67	27.88%	13.02
G_4_	166.13 ± 2.15^a^	179.22 ± 1.76	140.03 ± 2.78^b^	26.10	18.64%	13.09
G_5_	170.57 ± 3.29^a^	189.33 ± 1.45	137.57 ± 2.04^b^	33.00	23.99%	

Different letters in the same row indicate significant differences (*p* < 0.05).

As shown in Table 2, after five generations, the selected group had a significantly higher population density of 9075.00 ± 193.18 ind/L, consisting of 1330.33 ± 127.19 eggs, 5119.33 ± 233.68 nauplii, 1792.00 ± 184.46 copepodites, 460.67 ± 84.96 females, and 372.67 ± 43.73 males. In contrast, the control group had a lower population density of 7200.33 ± 249.18 ind/L, consisting of 1052.00 ± 189.06 eggs, 4392.33 ± 338.29 nauplii, 1185.00 ± 131.41 copepodites, 295.67 ± 119.92 females, and 275.33 ± 76.47 males. There was a significant difference in the number of copepodites between the selected and control groups (*p* < 0.05). There was no significant difference in intrinsic growth rate between the selected (0.55) and control groups (0.53). The sex ratio were assessed in the fifth generation and found that the ratio of female to male is 1.22 in the selected group and the ratio of female to male is 0.99 in the control group and the difference was not significant. However, there is no significant difference in the sex ratio between the selected and control group.

**Table 2:** Population numbers and intrinsic growth rates of the selected group and the control group across five generations of selection.

Group	Egg	Nauplii	Copepodite	Adult (Female)	Adult (Male)	Population (ind/L)	Intrinsic growth rate (R)
Selected	1330.33 ± 127.19^a^	5119.33 ± 233.68^a^	1792.00 ± 184.46^a^	460.67 ± 84.96^a^	372.67 ± 43.73^a^	9075.00 ± 193.18^a^	0.55
control	1052.00 ± 189.06^a^	4392.33 ± 338.29^a^	1185.00 ± 131.41^b^	295.67 ± 119.92^a^	275.33 ± 76.47^a^	7200.33 ± 249.18^b^	0.53

Data are presented as mean ± standard error. Different letters in the same column indicate significant differences (*p* < 0.05).

### Estimating the stability of the selection effect

**Fig. 7:**
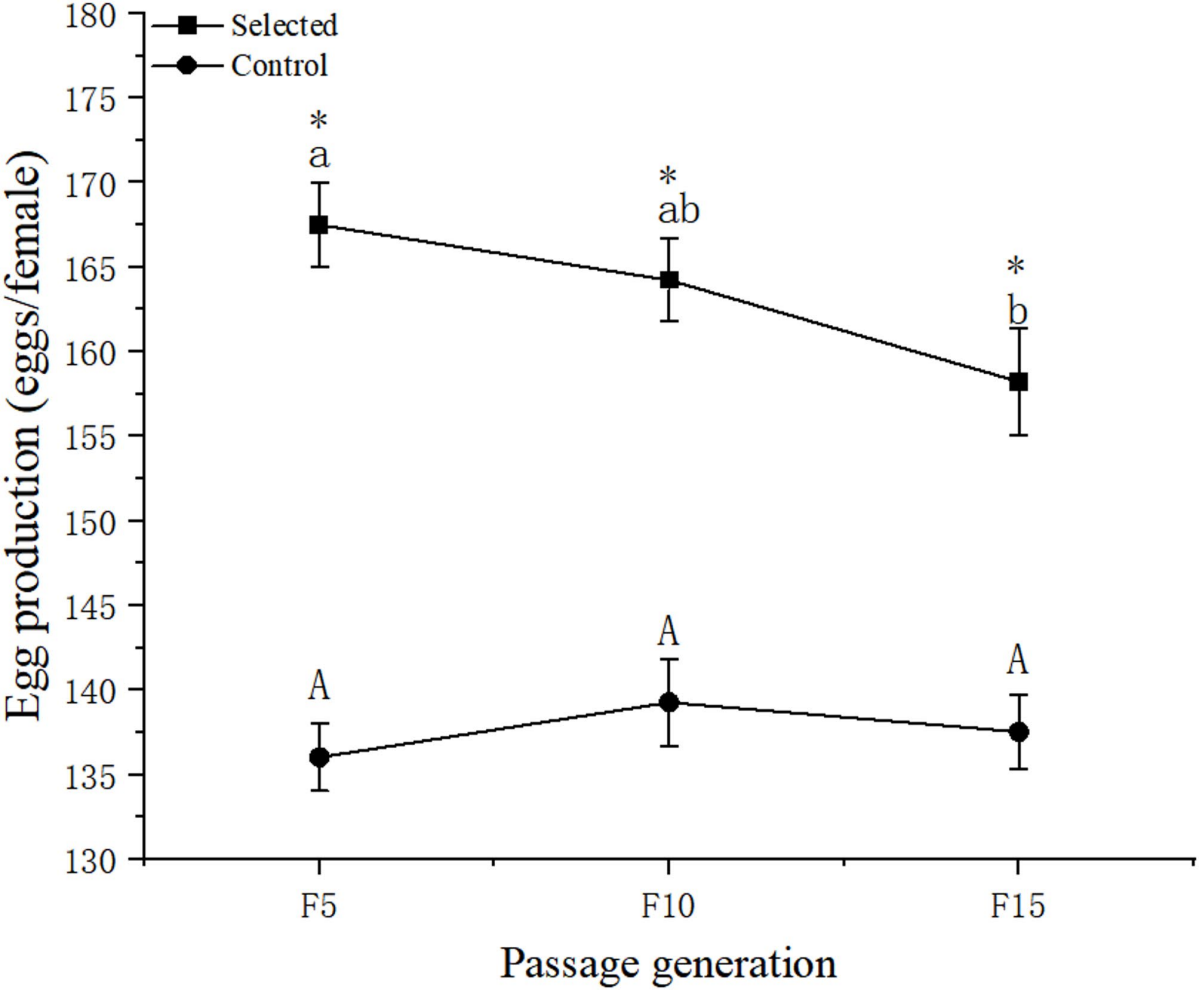
Total egg production (eggs/female) of the selected group and the control group after 5, 10, and 15 generations. Data are presented as mean ± standard error. The “*” indicates that a significant difference was observed between the selected and control groups. Different letters denote significant differences within the group.

Figure 7 displayed the total egg production of the selected group and the control group after 5, 10, and 15 generations. The total egg production of the selected group was significantly higher than that of the control group (*p* < 0.05). However, the total egg production of the selected group declined from 167.50 ± 2.47 eggs/female in the fifth generation to 158.23 ± 1.60 eggs/female in the 15th generation, which could be caused by inbreeding. In the 15th generation, the total egg production of the control group was 137.53 ± 2.28 eggs/female, which was 20.70 eggs/female less than that of the selected group.

After 5, 10, and 15 successive generations, there were significant differences in population density (all stages and post-embryonic stages) between the selected group and the control group. The population density (all stages) of the selected group decreased by 412.00 ind/L from the fifth generation to the 15th generation. In the 15th generation, the population density of the selected group was significantly higher than that of the control group, a difference of 1614.00 ind/L. The intrinsic growth rate (all stages and post-embryonic stages) of the selected group was significantly higher than that of the control group (Table 3).

**Table 3:** Population size and intrinsic growth rate after further propagation for 5, 10, and 15 generations.

Group	Generation	Population number (All stage)	Intrinsic growth rate (All stage)	Population number (Post embryonic stage)	Intrinsic growth rate (Post embryonic stage)
Control	F5	6857.67 ± 416.44^a^	0.53^a^	5817.00 ± 348.83^a^	0.52^a^
	F10	6707.00 ± 120.93^a^	0.53^a^	5774.67 ± 91.77^a^	0.52^a^
	F15	6809.00 ± 25.81^a^	0.53^a^	5867.67 ± 35.24^a^	0.52^a^
Selected	F5’	8835.00 ± 76.33^b^	0.55^b^	7796.33 ± 46.98^b^	0.54^b^
	F10’	8566.67 ± 93.24^b^	0.55^b^	7609.00 ± 69.51^b^	0.54^b^
	F15’	8423.00 ± 84.04^b^	0.55^b^	7384.00 ± 50.21^b^	0.54^b^

F represents the control group, and F’ represents the selected group. Data was presented as mean ± standard error. Different letters in the same column indicate significant differences (*p* < 0.05).

## Discussion

### Spawning characteristics of *Bestiolina amoyensis*

Egg production is a crucial metric for determining the productivity of copepods. Several factors, including temperature^41,42^, light intensity and photoperiod^35^, salinity^42^, and food^43^, influence egg production. This study builds upon previous findings conducted under optimal conditions. Wang et al.^35^ observed that the total egg production of *B. amoyensis* was 139.70 ± 4.40 eggs/female, with a hatching rate of 100%. Our results align with these findings. Both *B. amoyensis* and *P. crassirostris* belong to the family *Paracalanidae*. Similar to the results of *P. crassirostris* in Alajmi et al.^31^, the spawning curve of *B. amoyensis* also exhibited a parabolic shape with a slight decrease on the third day. Egg production peaked at 33.67 ± 2.30 eggs/female on the fourth day, indicating that *B. amoyensis* possesses strong reproductive and recovery capabilities, allowing it to rapidly adjust its reproductive strategy. The hatching rate is the foundation for evaluating the population recruitment. It was influenced not only by the maturity of fertilized eggs but also by the environmental conditions. In the present study, the experiments were conducted under the conditions described by Wang^35^, and it showed that the daily egg-hatching rate of *B. amoyensis* was close to 100%. High hatching success has also been reported for *Acartia bilobata*^26^, with rates approaching 100% when individuals were reared under optimal conditions. This further reflects that the present experiment was carried out under appropriate culturing conditions.

Study on *Paracartia grani*^44^ showed that food-limiting conditions significantly extended the survival duration of females and adult females could spawn almost to the ends of their lives, while spawning at much lower rates. On the contrary, under suitable conditions, the reproductive lifespan of this copepod was shortened, accompanied by a significant increase in total egg production. In present research, the spawning of *B. amoyensis* occurred within an 8-day window under suitable conditions. This pattern was consistent with findings for *A. bilobata*^26^, and comparable to that reported for *Oithona davisae* and *Temora longicornis*^45^.

Collectively, analysis of spawning performance not only demonstrated that our culture conditions were suitable but provided a reference for the formulation of subsequent selective breeding protocols.

### Breeding selection

Selective breeding has proven to be an effective method for enhancing desirable traits in copepods for aquaculture^46–48^. Our current study aimed to increase the total egg production of *B. amoyensis* through five generations of selection. The results revealed a significant increase in total egg production, with an average gain of 23.99% in the fifth generation, similar to previous findings by Alajmi^31^ and Santhanam et al.^33^. Moreover, in the present study, it reported that the reproductive lifespan of females was extended by 0.97 days after five generations of selection.

Alajmi et al.^31^ reported that the egg production (using production on days 3 and 4 pooled) could be improved by 24.50% through selective breeding; they observed that selected females continued spawning until day 8, while control line females ceased spawning on day 7. Santhanam et al.^33^ explored how temperature affected the reproductive capacity of *P. annandalei* through selective breeding, observing gains of 29.60% at 26 °C and 31.90% at 18 °C. This variation demonstrates the selective potential of fertility across different temperatures. Consistent with these studies, selective breeding also markedly improved total egg production and extended the reproductive lifespan of *B. amoyensis* females in present study, underscoring its potential to enhance the growth performance of *B. amoyensis*.

Egg production is often used as a key indicator of adult productivity and population growth, with high egg production suggesting increased offspring numbers and consequently the potential for population expansion^49,50^. Similar to prior research, our study found that selection significantly boosted the population size of *B. amoyensis*. The results showed the gender ratio in both the selected and control groups was approximately 1:1, while the selected group had a slight female bias. This gender ratio is crucial for reproductive capacity and population dynamics, suggesting that an increase in the proportion of females within the selected group could lead to rapid population growth. Additionally, it has been reported that the *B. amoyensis* exhibits a high intrinsic rate of population increase^35^, with value exceeding 0.5 under optimal conditions. In the present study, the intrinsic rates were 0.55 for the selected group and 0.53 for the control group, and there was no significant difference between these two groups. This further serves as indirect evidence that current selective strategy dose not impose adverse effects on the population growth dynamics of this species.

Heritability (ℎ^2^), as a critical measure of genetic variation in traits within a population, plays a significant role in understanding the response of a trait to selection^51^. The level of heritability determines the balance between genetic and environmental factors in trait development^52^. High heritability implies that a trait’s expression is predominantly governed by genetic rather than environmental factors^53^. Generally, a heritability greater than 0.4 reflects high heritability, and 0.2–0.4 indicates moderate heritability, while values below 0.2 indicate low heritability. In this study, the heritability of the total egg production of *B. amoyensis* indicated high heritability at 0.46. Alajmi et al.^31^ found that the heritability of the egg production of *Parvocalanus crassirostris* on the third and fourth days was 0.38. Santhanam et al.^33^ selected *Pseudodiaptomus annandalei* at two different temperatures, with a heritability of 0.64 at 18 °C and 0.30 at 26 °C for egg production. As shown in this article, a significant increase in total egg production within five generations selection and high heritability were observed, which indicated the trait of high fecundity would be improved by sustained selection.

### The stability of the selection effect

The selected individuals were propagated through generations 5, 10, and 15, to evaluate their reproductive ability and population density. The results indicated a gradual decrease in the total egg production of *B. amoyensis* for the selected group. Lee et al.^54^ observed that four generations of inbreeding did not negatively impact the development or lifespan of adult females of *Paracyclopina nana* Smirnov. However, in the eighth generation, inbreeding began to decrease individual fertility and population growth. The adverse effects of inbreeding not evident in early generations became progressively more apparent as inbreeding continued, leading to a significant decline in the vitality, productivity, reproductive ability, stress resistance, and adaptability of offspring^55,56^. The results of the present experiment were consistent with the above observations. In a confined space, the probability of inbreeding increases, leading to a decrease in the reproductive capacity of B. amoyensis and a weakened breeding effect.

Another reason for the decrease in egg production may be relaxation of selection, as the fitness of an allele depends on environmental conditions^57^. In the context of the cost of adaptation or trade-off, the loss and reversal of the acquired improvement in egg production were expected in the absence of artificial selection. This phenomenon has also been proven in copepods, the resistance of the *Acartia tonsa* to the dinoflagellate *Cochlodinium polykrikoides* could be increased after four generations of artificial selection. However, following a two-generation relaxation of selection, the elevated resistance was lost^58^.

Previous studies on *Pseudodiaptomus annandalei* have shown a significant population increase after five generations of selective breeding, with higher abundances of nauplii, copepodites, and adults compared to the control group^35^. In the present study, population density was measured after the 15th generation without selective breeding. While the selected group exhibited higher numbers of nauplii, copepodites, and adults than the control group, a statistically significant difference was observed only for copepodites. Additionally, populations dominated by nauplii typically reflected high female reproductive rates^59^. The maximum nauplii counts and proportions were observed in both the selected and control groups. Thus, given that a high reproductive rate was maintained after 15 generations. The high reproductive rate enabled the population to rapidly complete the life cycle from eggs to adults and ensured the sustained supply of larvae. Even if the adult population temporarily declined, the population structure could be quickly restored through the metamorphosis of a large number of larvae, thereby maintaining the relative stability of population dynamics. This was highly conducive to achieving the sustained supply of live feed, which rendered *B. amoyensis* a species with great potential for production application.

In conclusion, the present study provides the first evidence that selection can significantly enhance the reproductive capacity of *B. amoyensis* and maintain the advantage for at least 15 generations. The inbreeding problem is often been overlooked in the process of large-scale cultivation. Measures should be considered to mitigate the effects of inbreeding in future studies. It is advisable to periodically introduce individuals from an unselected population into the selected group to enhance genetic diversity, ensure sustained and stable large-scale production, and increase the algae concentration in the selected group to test whether this improves population stability and maintains the selection effect.

## Data Availability

The original contributions presented in the study are included in the article. Further inquiries can be directed to the corresponding author.
